# The Effects of Osteopathic Manipulative Treatment on Arrhythmias: A Double-blind Randomized Controlled Trial in Patients with Cardiac Implantable Electronic Devices

**DOI:** 10.19102/icrm.2024.15121

**Published:** 2024-12-15

**Authors:** Jacqueline Nikakis, Denis Malkov, Ermin Tale, Mahima Mangla, Jordan Keys, To Shan Li, Sheldon Yao, Todd J. Cohen

**Affiliations:** 1 Department of Clinical Specialties, New York Institute of Technology College of Osteopathic Medicine, Old Westbury, NY, USA; 2 Department of Medicine, Lenox Hill Hospital, Donald and Barbara Zucker School of Medicine at Hofstra/Northwell, New York, NY, USA

**Keywords:** Arrhythmia, autonomic nervous system, cardiac implantable electronic devices, heart rate variability, osteopathic manipulative treatment

## Abstract

This double-blind randomized controlled trial investigated the effects of osteopathic manipulative treatment (OMT) on cardiac arrhythmias in patients with cardiac implantable electronic devices (CIEDs). Participants (n = 41) with CIEDs were randomly assigned to either the OMT group or the control group (light touch/sham) and received a one-time intervention. No significant change in arrhythmia burden was found in the 1 month following intervention (*P* = .14). Discrete heart rate (HR), HR variability (HRV), and activity data were obtained from CIEDs in 17 of 41 subjects 1, 3, 7, 14, and 30 days prior to and after intervention. No significant difference was observed. An additional short-term substudy was performed on 20 subjects at the time of the intervention (5 min prior to and after intervention), and HR, respiratory rate, blood pressure, blood oxygen saturation (SpO_2_), and 1-min short-term HRV were compared. This study did not demonstrate an effect of OMT on arrhythmias, HR, respiratory rate, blood pressure, and blood SpO_2_. However, differences in OMT versus sham were observed for short-term HRV (*P* = .022) and a trend for long-term HRV. Importantly, there were no reported adverse effects with either intervention. OMT appears to be safe in cardiac patients.

## Introduction

Osteopathic manipulative treatment (OMT) is a hands-on approach used by osteopathic physicians to treat musculoskeletal restrictions that might affect the autonomic nervous system (ANS). Previously, the effects of OMT were investigated in healthy subjects in a randomized trial and found to have some influence on the ANS, with changes in heart rate variability (HRV) observed.^1^ The effects of OMT in patients with cardiac arrhythmias and cardiac implantable electronic devices (CIEDs) have not been studied before. Previously, we reported on the effects of OMT on the quality of life of the patients who were included in this study population.^2^ In this earlier analysis from our randomized controlled trial, we demonstrated a significant improvement in quality of life from OMT, specifically with respect to activities of daily living and pain, using the 36-Item Short Form Health Survey (SF-36). The purpose of this study was to examine the effect of OMT on cardiac arrhythmias and other physiologic variables in patients with CIEDs (both short and longer term).

## Methods

This double-blind randomized clinical trial (registered under ClinicalTrials.gov identifier NCT04004741) received approval from the institutional review board at the New York Institute of Technology College of Osteopathic Medicine with the reference number BHS-1464, focusing on the impact of OMT on arrhythmias. The investigation spanned from June 2019 to April 2022 and aimed to explore the effects of OMT on cardiac arrhythmias and other physiologic variables in patients with CIEDs.

Informed consent was obtained by the osteopathic neuromusculoskeletal medicine and osteopathic manipulative medicine (NMM/OMM) co-investigators at the time of enrollment before the procedure. Eligible participants who agreed to take part were randomly assigned to either the OMT group or the control group, receiving light touch (sham). The selection of OMT techniques by NMM/OMM board-certified co-investigators targeted autonomic input regions known to impact arrhythmias based on previous studies demonstrating effects on HRV.^1^

The inclusion criteria included age of ≥18 years, with a CIED, and documented arrhythmia-related complaints (ie, palpitations, shortness of breath, chest discomfort, presyncope/syncope). The exclusion criteria included active pregnancy, upcoming device changes, medication changes, prior spinal surgery, prior exposure to OMT, and contraindications to OMT.

The senior faculty member from the Department of Osteopathic Manipulative Medicine and the principal investigator/cardiologist were blinded to the subject randomization and were not present for the study administration. The study did not require external funding, and participants did not receive any compensation.

The subjects were randomized using Wolfram Mathematica 13.2 (Wolfram Research Inc., Champaign, IL, USA) and assigned numerical identifiers.^3^ All subjects then underwent an osteopathic structural examination. They received a single intervention (OMT or sham) administered by a board-certified NMM/OMM faculty member following a protocol based on established guidelines and reported in our study on quality of life in this same patient population.^2,4,5^ Techniques used included myofascial release, rib raising, facilitated positional release, and cranial osteopathic manipulative medicine **(Table 1)**. The light touch procedure (sham) was similar to that employed by Ruffini and colleagues in their HRV and OMM randomized trial.^1^ Specifically, the sham protocol entailed light touch applied in a specific sequence from the cranium down to the lower extremities. The duration of physician–subject contact was standardized across both study arms. A total protocol of 20 min was spent with each study participant, with 6 min allotted for osteopathic structural examination and 14 min allotted for OMT or sham.

**Table 1:** Osteopathic Manipulative Treatment Protocol

OMT Protocol	Light Touch Protocol
Rib raising/first rib FPR	Right ankle
Thoracic myofascial release	Left knee
Pectoralis lift	Right hip
Thoracic inlet release	Diaphragm
Cervical myofascial release	Right shoulder
Suboccipital release	Neck
Assessing for Chapman’s points	Cranium

*Abbreviation:* OMT, osteopathic manipulative treatment. The intervention group was treated with the above OMT techniques. The light touch treatment protocol. The control group was lightly contacted in these regions for 2 min each.

To account for variance in overall subject health, a baseline analysis of patients’ overall RAND SF-36 General Health score was performed using an unpaired Student’s *t* test (*P* ≤ .05) and was previously reported in our study assessing the effects of OMT in patients with CIEDs.^2^ At the 1-month follow-up visit, any changes in exercise, diet, or medications were noted.

The CIED data of the subjects were analyzed for arrhythmia burden 30 days prior to and after intervention. Significant arrhythmias included atrial fibrillation/atrial flutter (AF/AFl), supraventricular tachycardia (SVT) excluding AF/AFl, non-sustained ventricular tachycardia (NS-VT), and sustained VT/ventricular fibrillation. Discrete HR, HRV, and activity data obtained from the CIEDs of the subjects were analyzed at 1, 3, 7, 14, and 30 days prior to and after intervention.

Due to the coronavirus disease 2019 (COVID-19) pandemic, enrollment was paused between March 2020 and July 2021. Following reinitiation of the trial, a substudy was developed that investigated the short-term physiologic data of the subjects, including HR, respiratory rate (RR), blood pressure (BP), blood oxygen saturation (SpO_2_), and 1-min short-term HRV. These parameters were measured 5 min prior to and following intervention. HRV was measured using 1-min photoplethysmography and was calculated using the root mean square of successive R–R interval differences.

In addition to the physiologic variables measured, an additional substudy was performed to assess if light touch is an acceptable sham for randomized controlled trials with OMT. A survey was administered immediately after the intervention and asked subjects to reflect on which group they thought they had been allocated to: OMT, light touch, or if they were not sure.

All data are reported as mean ± standard deviation values. Statistical analysis included an unpaired Student’s *t* test; *P* ≤ .05 was considered statistically significant.

## Results

Forty-one subjects were enrolled in the study, with one lost to follow-up (22 OMT/18 sham). There were no reported adverse responses from either OMT or sham during the trial. At the 1-month follow-up, none of the subjects reported changes in their exercise, diet, or medications. Group differences in age, sex, and baseline SF-36 general health were analyzed and were not significantly different between the two groups. Types of CIEDs included 10 implantable loop recorders (ILRs), 9 pacemakers, and 3 implantable cardioverter-defibrillators (ICDs) in the OMT group and 9 ILRs, 5 pacemakers, and 4 ICDs in the control group, respectively **(Table 2)**.

**Table 2:** Subject Demographics

Subject Demographics	OMT (n = 22)	Control (n = 18)	*P* Value
Age, years	62.22 ± 16.39	62.72 ± 14.30	.920
Sex (male), %	50.00	27.78	.161
Preintervention general health^a^	51.20 ± 20.4	60.00 ± 31.2	.321
Type of CIED	10 ILR, 9 PM, 3 ICD	9 ILR, 5 PM, 4 ICD	—

^a^Pre-intervention scores from the SF-36 survey detailing general health.

*Abbreviations:* CIED, cardiac implantable electronic devices; ICD, implantable cardioverter-defibrillator; ILR, implantable loop recorder; PM, pacemaker; SF-36, 36-Item Short Form Health Survey.

### Arrhythmia data

**Figure 1:**
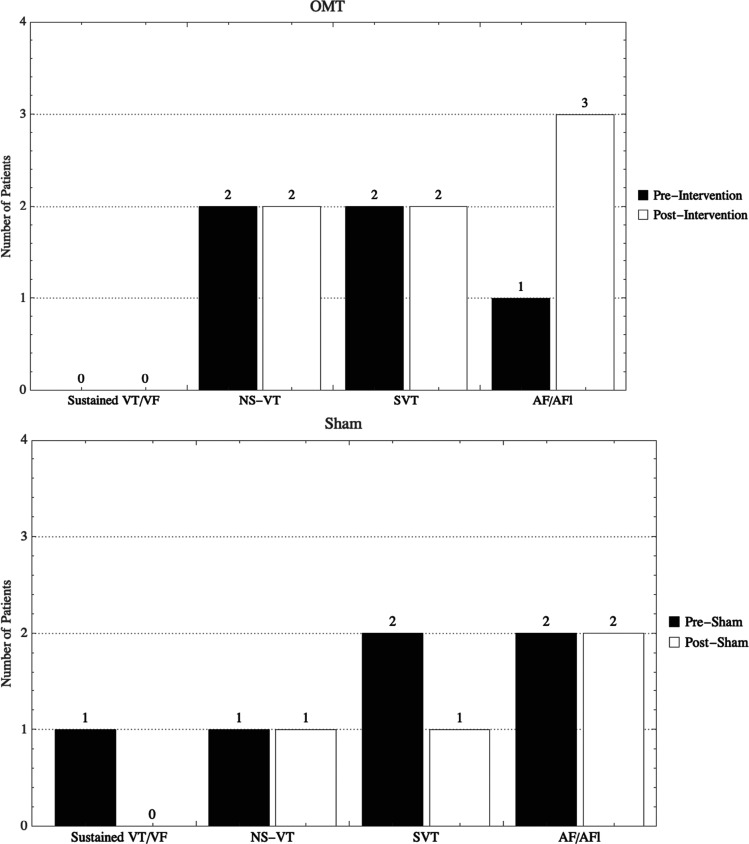
Change in arrhythmogenic events before and after intervention (osteopathic manipulative treatment or sham). *Abbreviations:* AF, atrial fibrillation; AFl, atrial flutter; NS-VT, non-sustained ventricular tachycardia; SVT, supraventricular tachycardia; VF, ventricular fibrillation; VT, ventricular tachycardia.

Arrhythmia data were available for all 40 retained subjects. The effects of OMT (n = 22) on arrhythmias were as follows: 5 increased, 5 decreased, and 12 had no change from prior to intervention **(Figure 1)**. The effects of light touch (n = 18) on arrhythmias were as follows: 5 decreased and 13 had no change. Between both groups, there was no significant change in arrhythmia burden following intervention (*P* = .14).

### Long-term physiologic data

**Figure 2:**
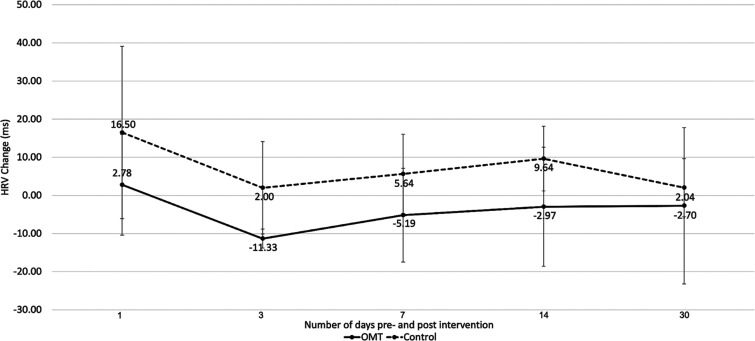
Short- and long-term changes in heart rate variability in the osteopathic manipulative treatment and control groups.

Discrete data were only available for devices actively enrolled in CareLink; therefore, the data were only available for 17 of the 41 subjects (10 OMT/7 control). **Figure 2** shows changes in physiologic measurements 1, 3, 7, 14, and 30 days prior to and after intervention. There was no statistically significant difference in changes in HR, HRV, and daily activity between the groups.

### Short-term physiologic data

Twenty subjects were enrolled (10 OMT/10 control) in our substudy, analyzing the short-term physiologic effects of OMT in CIED patients; changes in HR, RR, BP, and SpO_2_ were no different between groups measured 5 min prior to and following intervention. The baseline average HRVs for the OMT group and the control group were 42.63 ± 37.24 ms and 35.92 ± 24.80 ms, respectively. Additionally, postintervention HRV averages were 36.66 ± 20.27 ms and 55.86 ± 34.18 ms for the OMT and control groups, respectively. The HRV of the OMT group decreased by 12.39 ± 34.05 ms, whereas the HRV of the control group increased by 19.94 ± 18.54 ms (*P* = .022) **(Table 3)**.

**Table 3:** Short-term Physiologic Results in the Osteopathic Manipulative Treatment and Control Groups

Parameter	OMT (n = 10) (Mean ± SD)	Control (n = 10) (Mean ± SD)	*P* Value
Age, years	62.70 ± 16.64	64.70 ± 17.67	.80
Sex (male), n (%)	5 (50.00)	5 (50.00)	1.00
Type of CIED	5 ILR, 4 PM, 1 ICD	5 ILR, 4 PM, 1 ICD	1.00
ΔHR, bpm	−4.00 ± 5.83	−4.90 ± 8.17	.79
ΔRR, bpm	−2.70 ± 3.10	−0.60 ± 3.69	.20
ΔMAP, mmHg	−1.77 ± 4.13	0.53 ± 6.38	.38
ΔSpO_2_, %	0.00 ± 0.02	0.01 ± 0.01	.69
ΔHRV, ms	−12.39 ± 34.05	19.94 ± 18.54	.022*

*Abbreviations:* HR, heart rate; HRV, heart rate variability; ICD, implantable cardioverter-defibrillator; ILR, implantable loop recorder; MAP, mean arterial pressure; PM, pacemaker; RR, respiratory rate; SD, standard deviation; SpO_2_, oxygen saturation measured by pulse oximetry. *Statistical significance, *P* < .05.

### Placebo analysis

The same 20 subjects were enrolled in our substudy (10 OMT/10 control) to assess whether the sham approach was an effective control for OMT. Four (40.0%) subjects in the OMT arm and five subjects (50.0%) in the sham arm correctly perceived their randomization (*P* = .25).

## Discussion

To our knowledge, this is the first randomized clinical trial to provide insight into the effects of OMT in patients with CIEDs. Previously, our team reported that a single OMT intervention in this same cohort had significant effects on quality of life, specifically on activities of daily living and pain reduction.^2^ Our current study found no significant effect on arrhythmias and most physiologic variables (except for HRV during a short-term substudy) in patients with CIEDs. A single treatment appeared ineffective at modulating arrhythmias in this preliminary study; however, more frequent treatment sessions might have a different outcome. Additional studies are necessary to determine whether OMT may impact arrhythmias as well as other physiologic variables in a different capacity. Perhaps the application of physical touch can support beneficial physiologic outcomes and impact cardiac function. This raises an important question on the impact of OMT versus touch on patients’ health outcomes. Our study began to address this question with patients. Notably, this study did not demonstrate any adverse responses to OMT or light touch/sham. This was also highlighted by the fact that neither intervention appeared to have any pro- or anti-arrhythmic effects in patients with CIEDs. OMT appears to be a safe adjunctive therapy in patients with cardiac arrhythmias and CIEDs, including ILRs, pacemakers, and ICDs.

When investigating whether light touch served as an effective control for OMT in our substudy, subjects could not correctly identify their randomization group half of the time. This finding validated the sham group as an effective control for OMT in this randomized clinical trial.

Autonomic dysfunction has been implicated in the pathogenesis of arrhythmias in those with chronic pain.^6,7^ Specifically, those with overactivation of the sympathetic nervous system have an increased risk of AF and ventricular arrhythmias.^6–9^ Previous studies have demonstrated that OMT may influence the ANS in many patient populations.^10^ As an indirect proxy for ANS function, HRV may provide some utility in evaluating the effectiveness of OMT in conditions characterized by ANS imbalance. In particular, HRV measurements have served as proxies for cardiac vagal function.^10^ Importantly, HRV was measured in two different ways in our study: 1-min short-term photoplethysmography (short-term study) and 1-, 3-, 14-, and 30-day discrete measurements obtained by CIEDs using electrograms (long-term study). Short-term HRV decreased significantly in the OMT group following intervention (*P* = .022; **Table 3**). On close examination of long-term physiologic data, although HRV did not change significantly at any time, the OMT group appeared to have a decrease in HRV at 3 days (−11.33 ± 12.13 ms; *P* = .07) and 14 days (2.97 ± 8.50 ms; *P* = .08), similar to that observed with short-term HRV **(Figure 2)**. No other statistically significant differences or trends were observed in long-term changes in HR, HRV, and activity levels between the groups **(Table 4)**.

**Table 4:** Short-term and Long-term Physiologic Results in the Osteopathic Manipulative Treatment and Control Groups.

	±1 Day			±3 Days			±7 Days			±14 Days			±30 Days		
	OMT Group	Control Group	*P* Value	OMT Group	Control Group	*P* Value	OMT Group	Control Group	*P* Value	OMT Group	Control Group	*P* Value	OMT Group	Control Group	*P* Value
Heart rate variability, ms	2.78 ± 22.56	16.5 ± 13.2	.28	−11.33 ± 12.13	2.00 ± 2.52	.07	−5.19 ± 10.42	5.64 ± 12.32	.13	−2.97 ± 8.50	9.64 ± 15.6	.08	−2.70 ± 7.59	2.04 ± 20.46	.54
Night heart rate, bpm	−3.9 ± 7.62	−1.4 ± 2.1	.49	−1.83 ± 5.71	−0.22 ± 1.33	.51	−0.8 ± 2.89	0.37 ± 2.35	.42	−0.28 ± 3.54	−0.66 ± 2.55	.82	−0.53 ± 3.26	0.26 ± 3.31	.63
Day heart rate, bpm	−0.65 ± 2.47	−0.33 ± 3.14	.83	−1.64 ± 4.0	−1.67 ± 4.32	0.72	−1.20 ± 1.87	−1.96 ± 3.10	.55	−0.63 ± 2.13	−1.96 ± 2.89	.31	0.09 ± 2.0	−13.78 ± 26.6	.12
Weighted average heart rate, bpm	−1.46 ± 2.91	−0.08 ± 2.52	.35	−1.69 ± 2.52	−1.21 ± 3.42	.52	−1.10 ± 1.79	−1.38 ± 2.51	.80	−0.54 ± 2.32	−1.40 ± 2.01	.44	−0.07 ± 1.98	−10.27 ± 20.21	.13
Daily activity level, min	−8.7 ± 58.6	35.17 ± 48.0	.15	14 ± 31.9	18.5 ± 28.48	.79	−3.36 ± 24.67	3.02 ± 12.83	.57	−8.18 ± 18.68	−4.63 ± 14.73	.70	−0.69 ± 13.74	−4.56 ± 12.24	.56

Results are presented as mean ± standard deviation values.

### Limitations

Due to the COVID-19 pandemic, enrollment was paused between March 2020 and July 2021. Patient intervention was difficult, if not impossible, during parts of the pandemic, and some longer-term data were not available due to issues with remote monitoring and patient loss to follow-up, particularly long-term physiologic data from the entire subject cohort.

Additionally, this study investigated the effects of only a single intervention (OMT vs. sham). This study design was limited to the flexibility of the subject clinical follow-up. Future randomized clinical trials should be performed in a larger cohort to corroborate the results. The effects of multiple interventions, such as weekly sessions, should be studied to assess the impact of more frequent OMT treatment on arrhythmias and possibly determine an optimal treatment schedule. Furthermore, a future study with a three-arm design—OMT, sham, and time control—may be considered to further limit and account for any placebo effect.

## Conclusions

This is the first randomized controlled trial to investigate the short- and long-term effects of OMT on physiologic parameters and arrhythmias in patients with CIEDs. This study did not demonstrate an effect of OMT on arrhythmias, HR, RR, BP, or SpO_2_. However, differences in OMT versus sham were observed for short-term HRV and a trend for longer-term HRV. Importantly, there were no reported adverse effects in either intervention. OMT appears to be safe in cardiac patients.
